# Supplementary material for: A high-resolution mRNA expression time course of embryonic development in zebrafish
Source: eLife. 2017 Nov 16;6:e30860. doi: 10.7554/eLife.30860 (PMC5690287; doi:10.7554/eLife.30860)
Supplement: Supplementary file 6. [file elife-30860-supp6.zip › biolayout-clusters-files/Cluster248-genes.html]

Cluster248


# Cluster248: Genes

| Ensembl ID | Gene Name | Chr | Start | End | Biotype |
| --- | --- | --- | --- | --- | --- |
| ENSDARG00000102483 | ENSDARG00000102483 | 15 | 43997275 | 44028409 | protein\_coding |
| ENSDARG00000075883 | bub3 | 12 | 49193302 | 49197913 | protein\_coding |
| ENSDARG00000031680 | ercc1 | 9 | 32863930 | 32873262 | protein\_coding |
| ENSDARG00000023989 | pef1 | 19 | 37533371 | 37547091 | protein\_coding |
| ENSDARG00000035427 | surf6 | 5 | 32674997 | 32681003 | protein\_coding |
| ENSDARG00000019406 | zgc:63470 | 7 | 620155 | 626604 | protein\_coding |
